# Neonatal cerebral ultrasound: anatomical variants and age-related diseases

**DOI:** 10.1007/s40477-024-00914-8

**Published:** 2024-06-25

**Authors:** Giulia Fichera, Roberto Stramare, Gianni Bisogno, Rolf Wyttenbach, Barbara Simonetti Goeggel, Filippo Del Grande, Chiara Giraudo, Marirosa Cristallo Lacalamita

**Affiliations:** 1 Pediatric Radiology, University Hospital of Padova, Padua, Italy; 2 Department of Cardiac, Thoracic, Vascular Sciences and Public Health (DCTV), University of Padova, Padua, Italy; 3 Pediatric Hematology-Oncology Division, University Hospital of Padova, Padua, Italy; 4 Imaging Institute of Southern Switzerland EOC, Bellinzona, Switzerland; 5 Faculty of Biomedical Sciences, Università Della Svizzera Italiana, Lugano, Switzerland; 6 Department of Neurology, University Hospital Bern, University of Bern, Bern, Switzerland; 7 Department of Neuropaediatrics, Institute of Paediatrics of Southern Switzerland, San Giovanni Hospital, Bellinzona, Switzerland; 8 University of Zurich, Zurich, Switzerland; 9 Department of Radiology, Ospedale Regionale di Lugano, Lugano, Switzerland; 10 Department of Diagnostic, Interventional and Pediatric Radiology (DIPR), Inselspital, Bern University Hospital, University of Bern, Bern, Switzerland

**Keywords:** Cerebral ultrasound, Infants, Anatomical variants, Brain disorders

## Abstract

Cerebral ultrasound is a non-invasive imaging technique widely used for the assessment of brain anatomy and diseases in neonates and infants. Indeed, it allows a precise characterization of common variants such as cavum septum pellucidum or diseases like intraventricular hemorrhage. The aim of this pictorial review is to provide a comprehensive overview of the main ultrasound features of the most common cerebral anatomical variants and disorders detectable by cerebral ultrasound using an age-related approach which could support non-subspecialized radiologists.

## Introduction

Cerebral ultrasound (cUS) is a non-invasive imaging technique, easily performed up to the third month of life, that allows detection of brain abnormalities in neonates and infants. cUS can be performed at bedside being particularly useful in neonatal intensive care units and emergency departments where a quick assessment is necessary. Moreover, cUS provides crucial information regarding the evolution of lesions [1].

This pictorial review, including a short technical description of cUS, provides a comprehensive overview of the main ultrasound features of the most common cerebral anatomical variants and disorders detectable by cUS applying an age-related disease classification: (i) premature infants; (ii) term newborns; and (iii) older infants.

## Anatomical variants

**Fig. 1:**
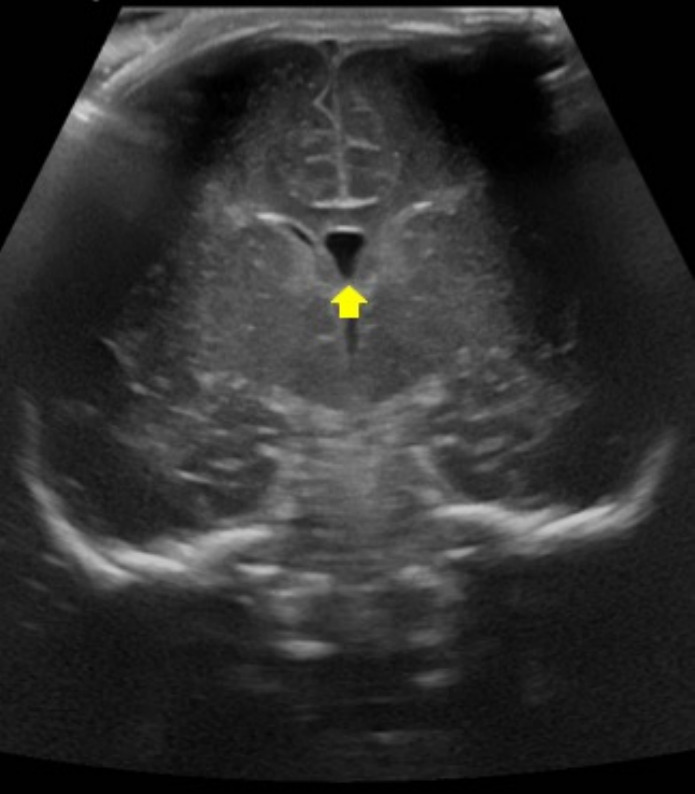
Cerebral ultrasound of 1-day-old premature male born at 32 weeks and 4 days of gestation showed midline fluid-filled cyst between the lateral ventricles on the coronal plane (yellow arrow) consisting in common anatomical variant such as Cavum Septum Pellucidum

Cerebral variants can be commonly identified by imaging and should not be misinterpreted as pathological findings. Often the variants affect the midline, like for instance the cavum septum pellucidum, cavum vergae, and the cavum veli interpositi. A frequent variant of the posterior cranial fossa is the mega cisterna magna, while off-midline changes include choroid plexus and connatal cysts. The cavum septum pellucidum and the cavum vergae are midline small fluid-filled cysts between the lateral ventricles usually ranging from 2 to 10 mm [1]. The former is located anteriorly to the Monro foramen while the cavum vergae is posterior (Fig. 1) [2]. Both variants occur in around 100% of preterm neonates while the cavum septum pellucidum and the cavum vergae are identified in 80% and 30% of term births, respectively [3].

**Fig. 2:**
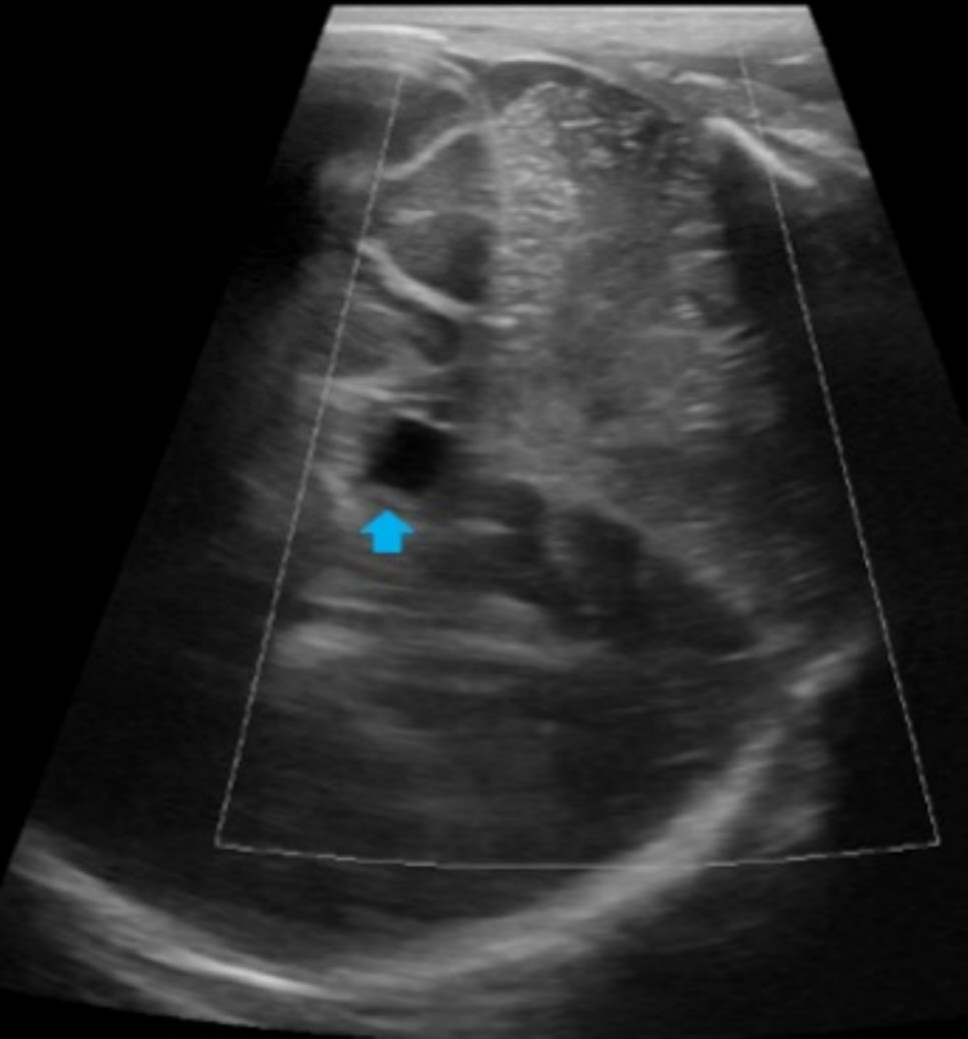
Cerebral ultrasound of 2-day-old full term male showed cavum veli interpositi, appearing as an interhemispheric well-defined unilocular anechoic cyst on mastoid view (blue arrow)

The cavum veli interpositi is a rare variant characterized by an interhemispheric well-defined unilocular anechoic cyst, anteroinferior to the splenium of the corpus callosum, and superior and posterior to the thalami [2] (Fig. 2).

**Fig. 3:**
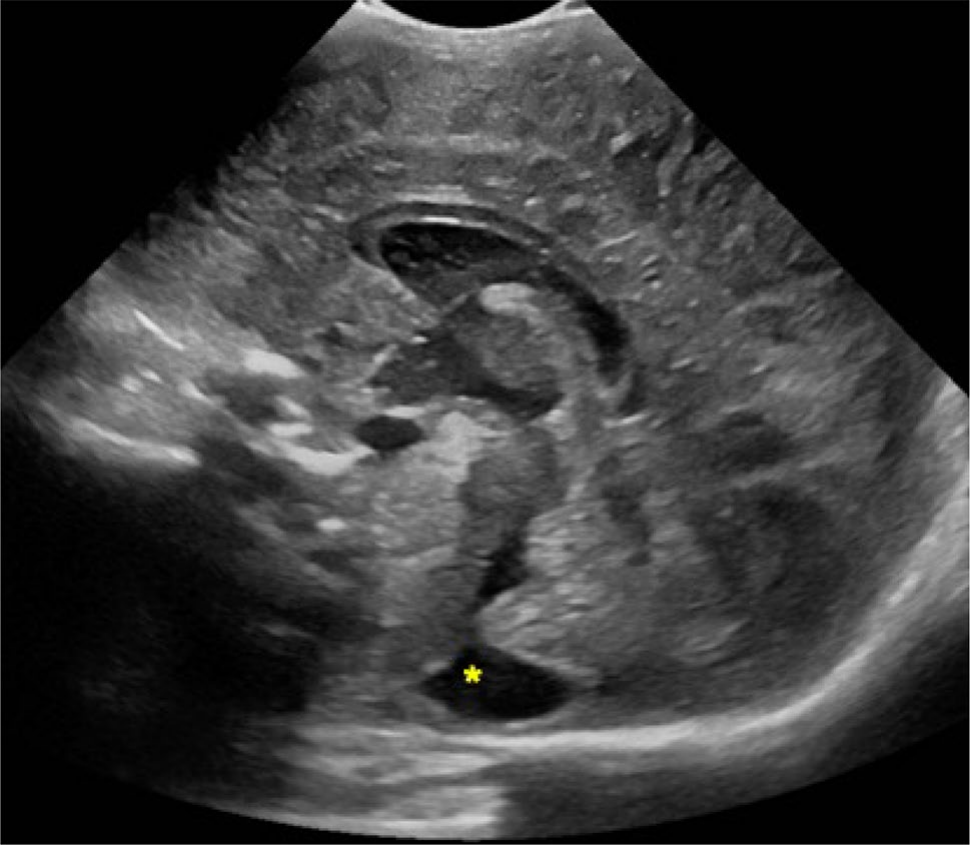
Screening cerebral ultrasound of a premature female born at 32 weeks and 6 days of gestation showing on sagittal view well-defined anechoic space located in the posterior cranial fossa (yellow asterisk). This finding was suggestive of mega cisterna magna (common anatomical variant)

The mega cisterna magna is a cerebrospinal fluid-filled space, in the posterior cranial fossa, communicating with the fourth ventricle. A sagittal plane is recommended to better visualize the well-defined anechoic space greater than 10 mm which extends from the foramen magnum to the caudal margin of the inferior vermis. The cerebellar vermis and the ventricular caliber are normal [1, 4] (Fig. 3).

**Fig. 4:**
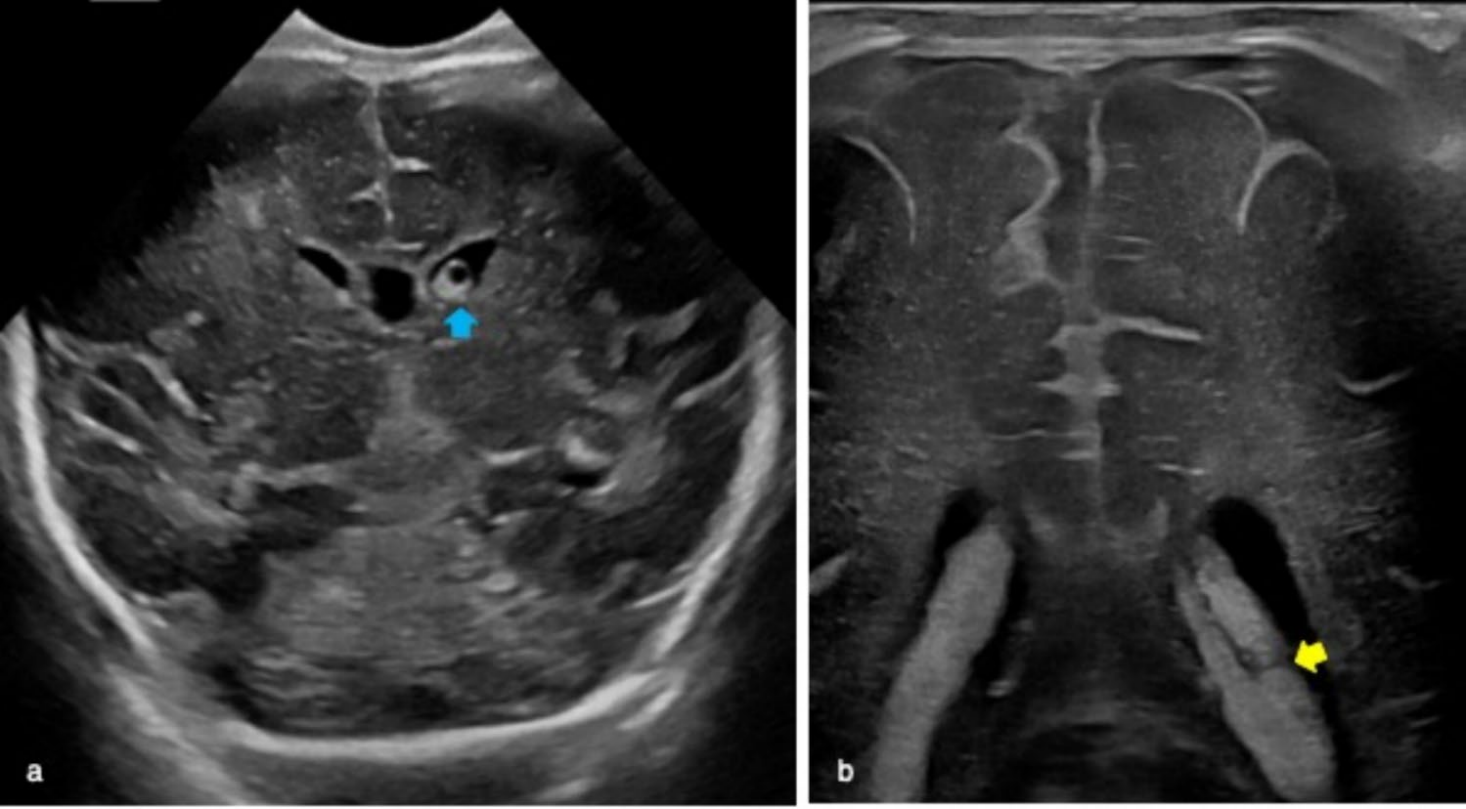
Coronal planes of cerebral ultrasound performed on premature infant demonstrated left well-defined cyst (blue arrow in **a**) and hyperechoic central cleft defining split choroid sign (yellow arrow in **b**). These are typical findings of choroid plexus cyst

The choroid plexus is located within the cerebral ventricles and it consists of a network of blood vessels covered by a layer of ependymal cells producing cerebrospinal fluid. The glomus is its largest part and sometimes may present a central cleft (aka “split choroid sign”; Fig. 4). This finding may mimic a choroid haemorrhage and the use of Color Doppler supports their differentiation [1]. Unilateral small cysts of the choroid plexus (diameter < 10 mm) are common while the occurrence of multiple cysts is usually associated with chromosomal diseases.

**Fig. 5:**
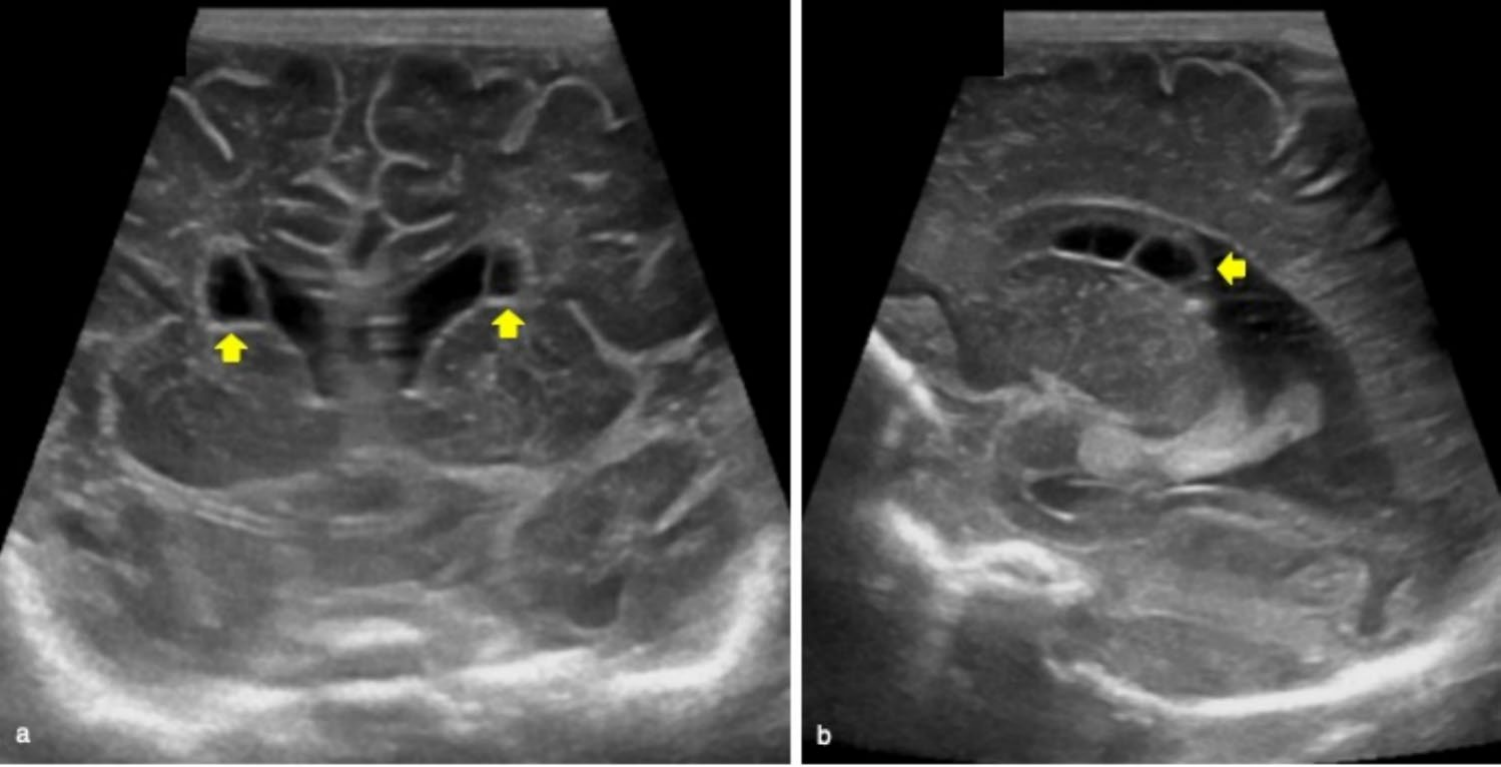
Cerebral ultrasound of 4-day-old full-term male well demonstrating bilateral multiple-rounded cysts located in the frontal horns of the lateral ventricles on coronal plane (yellow arrows in **a**) with the typical “string-of-pearls” appearance on sagittal view (yellow arrow in **b**). These findings are suggestive of connatal cysts

Connatal cysts are bilateral symmetric multiple-rounded cysts located in the superolateral angles of the frontal horns of the lateral ventricles with the typical “string-of-pearls” appearance at cUS [4] (Fig. 5).

## Premature infants

The most frequent brain disorders occurring in premature infants (i.e., infants born alive before completion of the 37th week of gestation) include germinal matrix-intraventricular hemorrhages and periventricular leukomalacia [5].

### Germinal matrix and intraventricular hemorrhage (GM-IVH)

GM-IVH is a common condition in premature infants, with very low birth weight, frequently occurring during the first week of life and cUS is the modality of choice to early detect and define its severity [6]. In the early phase, hemorrhages may appear as well-defined hyperechoic spots in the subependymal germinal matrix extending along the caudothalamic groove. If bleeding persists it involves the lateral ventricles leading to intraventricular hemorrhages.

**Fig. 6:**
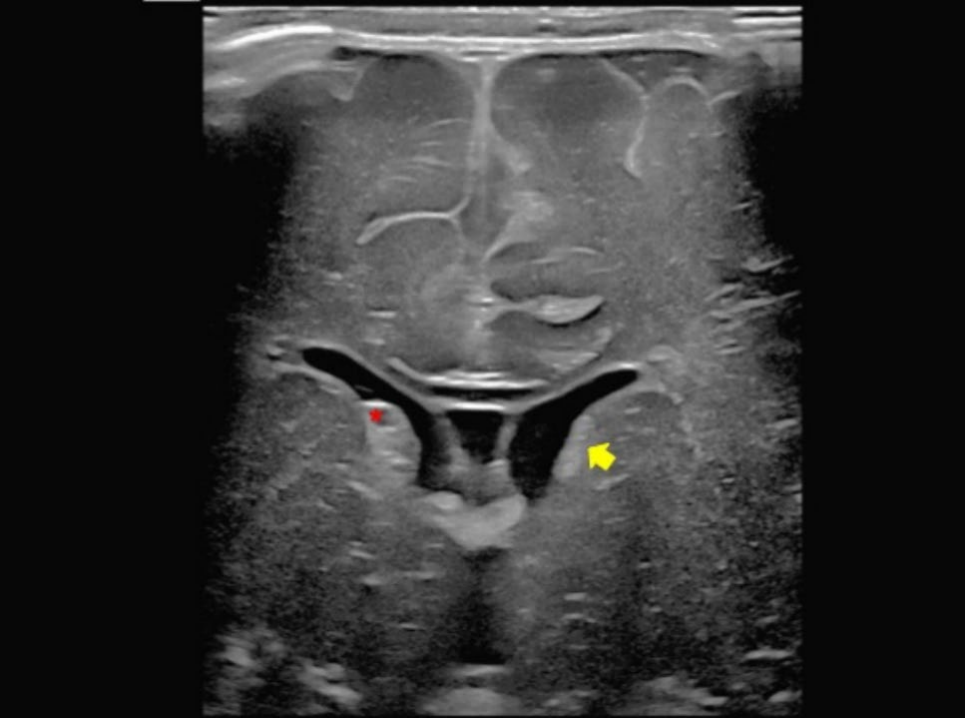
Premature male infant born at 34 weeks’ gestation who underwent cerebral ultrasound at 2-month old for fever (up to 38.7 °C) and anemia. Coronal plane well showed bilateral hemorrhage in the groove between the thalamus and the nucleus caudate. Grade 1 on the left side and grade 2 on the right side (yellow arrow and red asterisk, respectively)

Two different scores (i.e., the Papile score adapted for US and the Volpe grading system) [7, 8] have been proposed to grade GM-IVH and both suggest four grades of severity. In particular in grade 1 hemorrhages are limited to the germinal matrix without any extension in the ventricle or with less than 10% of involvement [9] (Fig. 6). In grade 2 the intraventricular hemorrhage ranges between 10 and 50% but without any ventricular dilatation [9] (Fig. 6).

**Fig. 7:**
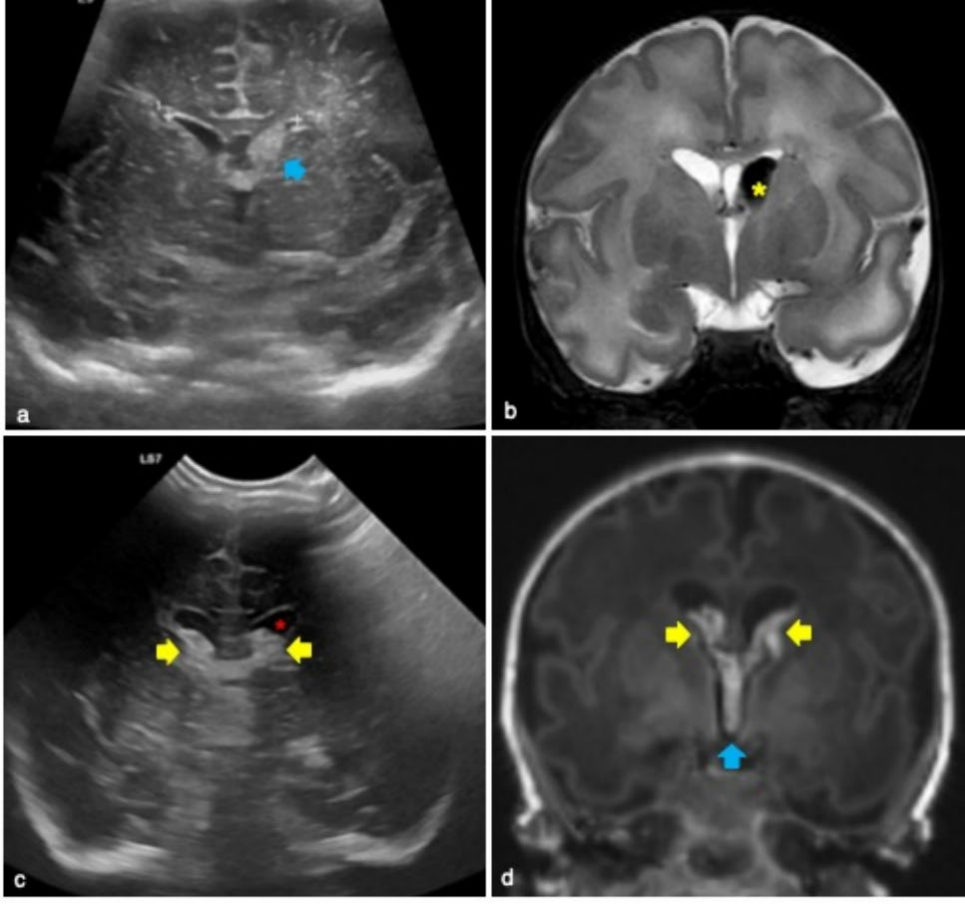
Typical findings of germinal matrix hemorrhage of grade 3. Premature male infant born at 34 weeks’ gestation who underwent cerebral ultrasound at 4 days of life showing left subependymal germinal matrix hemorrhage of grade 3 on coronal plane (blue arrow in **a**). This finding was confirmed on T2 weighted image by MR (yellow asterisk in **b**). Premature male infant born at 33 weeks and 5 days gestation who underwent cerebral ultrasound at first day of life showing both lateral ventricular hemorrhages and ventriculomegaly on coronal plane (yellow arrows and red asterisk in **c**, respectively). Magnetic Resonance performed to complete evaluation, confirmed intraventricular and third ventricle hemorrhages on coronal T1 weighted (yellow and blue arrows in **d**, respectively)

In grade 3 the intraventricular hemorrhage involves more than 50% of the lateral ventricle (Fig. 7). Grade 4 is characterized by a parenchymal hemorrhagic infarction. In fact the dilatated ventricle filled with blood may cause compression of the deep terminal veins which in turn lead to venous infarction. In these patients a mass effect with shift of the midline may occur.

**Fig. 8:**
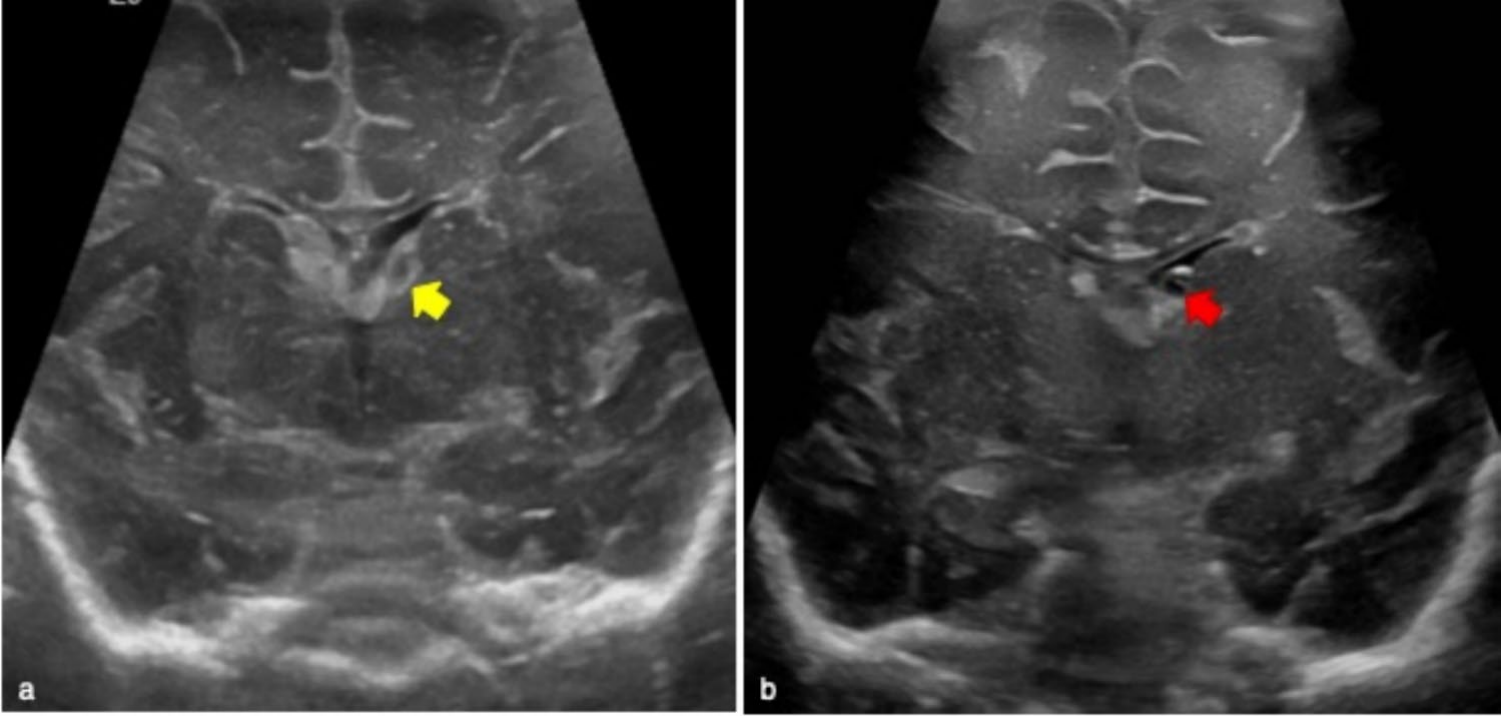
Follow-up ultrasound of premature male infant born at 34 weeks gestation demonstrated progressive evolution of grade 2 left subependymal germinal matrix hemorrhage. In a intermediate phase of colliquation (yellow arrow in **a**), followed by subependymal cyst evolution after 2 weeks (red arrow in **b**)

In some days or weeks, grade 1 and 2 hemorrhages are replaced by colliquation and then by subependymal cyst (ranging from 3 to 5 mm). The complete resolution occurs in 8 weeks (Fig. 8). Grades 3 and 4 usually take some weeks to improve and there might be long-term neurocognitive sequelae, including post-hemorrhagic hydrocephalus, cerebral palsy and disabilities in around 50–75% of patients [10].

### Periventricular leukomalacia (PVL)

**Fig. 9:**
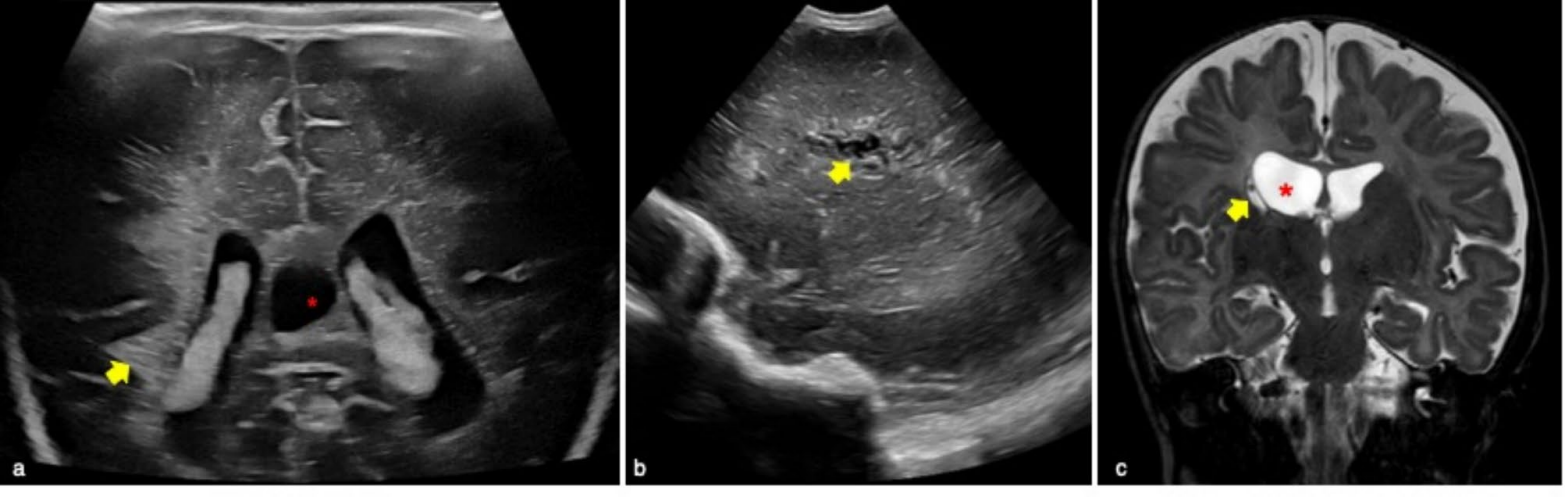
Early and late phases evolutions of periventricular leukomalacia of premature infants. **a** Third-day of life cerebral ultrasound of premature male infant born at 31 weeks and 5 days gestation showed right periventricular white matter hyperechoic areas (yellow arrow), particularly in the right occipital horn. These findings are typical of first 48-h periventricular leukomalacia. Cavum Vergae as anatomical variant is also showed (red asterisk in **a**). Follow-up radiological imaging of premature female infant born at 33 weeks gestation and birth weight of 1300 g performed three months after first ultrasound examination (not showed). Sagittal ultrasound plane well demonstrated cystic changes of the periventricular white matter (yellow arrow in **b**). Cystic areas were confirmed on coronal MR T2 weighted, also well demonstrating right ventricular dilatation (yellow arrow and red asterisk in **c**, respectively). These were typical findings of late-phase evolution of periventricular leukomalacia

PVL is the most common form of cerebral white matter injury secondary to ischemia in preterm infants. It is frequent in case of < 32 weeks gestational age and/or weight < 1500 g at birth with an estimated incidence of 7.7% [11]. The pathogenesis of PVL is related to ischemic phenomena in the periventricular watershed zones adjacent to the trigones of the lateral ventricles and to the foramina of Monro [9]. PVL features at cUS change with time. In fact in the first 48 h, periventricular hyperechoic areas are usually seen [9] (Fig. 9). In first 2–4 weeks of life there might be a transient period of brain tissue normalization [9, 12].

Three to six weeks later, frontoparietal small cysts ranging from a few millimeters to over a centimeter could be present (Fig. 9). Then, around six months, the cysts disappear and there might be a loss of periventricular white matter and ventricular enlargement [13].

## Full-term newborns

Cerebral diseases in full-term newborns typically occur around from the 37th to 42nd week of gestation.

### Hypoxic-ischemic encephalopathy (HIE)

HIE is secondary to perinatal asphyxia and it occurs in around 3 to 6/1000 live births [14]. Antepartum (i.e., maternal hypotension, infertility treatments, infections), intrapartum (i.e., use of forceps, abruptio placentae, prolapse of the umbilical cord, and uterine rupture) or postpartum disorders (i.e. severe respiratory distress, sepsis, and shock) may induce HIE. On the first days of life, cUS could be negative with a sensitivity of 50% [15]. MR, especially including Diffusion Weighted Imaging (DWI), represents the gold standard for ischemic lesions characterizing the peak of the extent of brain injury after 3 to 5 days (Fig. 11). cUS is often used during follow-up to monitor the echogenicity of the brain and its vascularization [14].

**Fig. 10:**
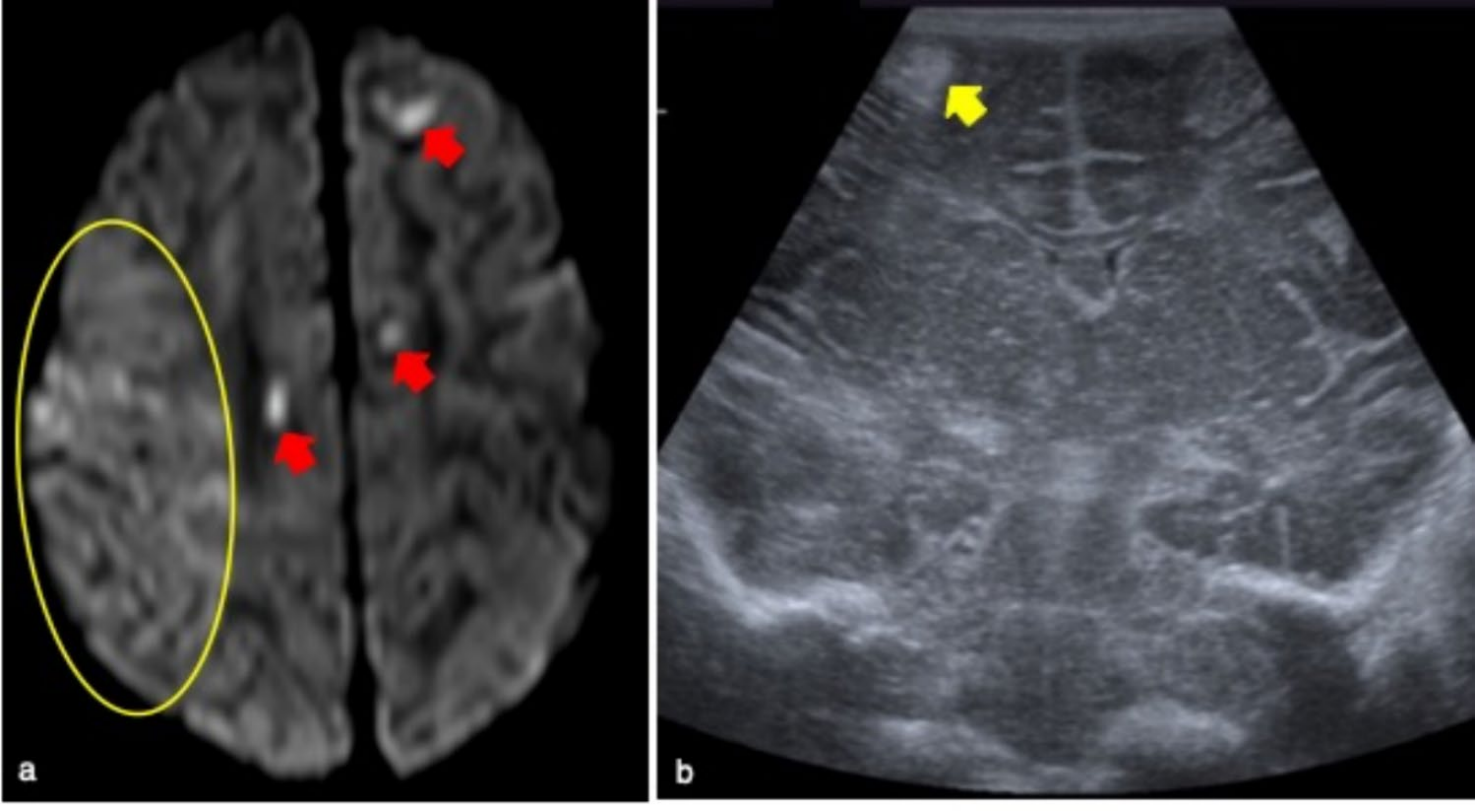
Full term female infant who underwent cerebral ultrasound at 3-days of life for central apneas and epileptic seizures. No cerebral pathological findings were identified on first ultrasound evaluation (not showed), therefore MR was performed at day 4 of life to detect brain anomalies. Right-side acute ischaemia of the temporal lobe and multiple cortical petechiae were detected on DWI (yellow circle and red arrows in **a**, respectively), which represent typical findings of hypoxic-ischemic encephalopathy. Follow-up cerebral ultrasound showed a corticalfocal hyperechoic area on coronal view as outcome of ischaemia (yellow arrow in **b**)

Ischemic injuries usually occur in the border zones between the anterior and the middle cerebral artery or between the middle and the posterior ones [13]. If HIE is detected by cUS, it can be classified as mild, moderate, or severe. In the mild grade there is a slightly narrowed lateral ventricle with normal brain parenchyma. In moderate anoxic events diffuse or focal parenchymal hyperechoic areas in the cortex and in the subcortical white matter can be identified (Fig. 10).

Severe HIE are characterized by areas of hyperechogenicity in the gray matter (e..g, basal ganglia and thalami), decreased blood flow and increased resistive index on Color Doppler. Ventriculomegaly and cerebral hemorrhage may also occur [13, 14]. Neonates with severe HIE, monitored for 12 months, had sequelae such as ventricular dilatation and brain atrophy [13].

### Neonatal arterial ischemic stroke (NAIS)

NAIS can occur in full-term newborns, typically within the first 28 days of life with an incidence of 1 case in 5000 live births [16]. The main risk factors include male sex, primiparity, physiological hypercoagulability of the newborn, thrombophilia, placental pathologies, perinatal hypoxia, and fetal/neonatal infections [16].

**Fig. 11:**
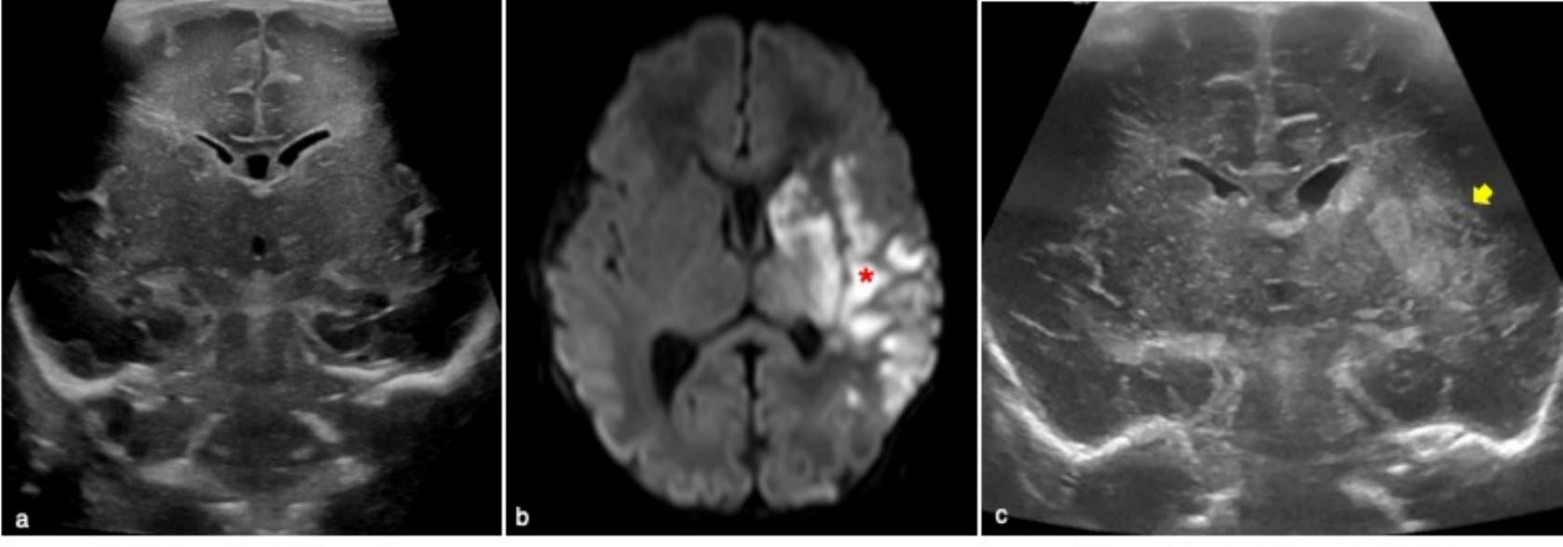
Full term male infant who underwent cerebral ultrasound at second day of life for central apneas and convulsive seizures. No pathological findings were detected on cerebral ultrasound (**a**). Due to symptoms, MR was also performed, showing an extensive ischaemic lesion in the middle cerebri artery territory on DWI (red asterisk in **b**). The same patient underwent a follow-up cerebral ultrasound after one week from acute event, well demonstrating a left cortico-subcortical hyperechoic area (yellow arrow in **c**)

In the early phase, cUS is often normal but this technique supports the diagnosis of abnormalities later on. The middle cerebral artery is frequently affected and therefore the zones receiving its vascular supply may present loss of grey-white matter differentiation and mild mass effect followed by wedge-shaped focal hyper-echoic areas [2] (Fig. 11). In case of severe asphyxia low resistive indexes (< 0.6) are idenfied at Color Doppler [1]. The typical findings of the late phase (after 2–6 weeks) include ventriculomegaly and enlargement of interhemispheric fissures. Even if cUS can contribute to the diagnosis of NAIS, it should not be overlooked that MR is the gold standard [17].

### Neonatal cerebral sinovenous thrombosis (CSVT)

Venous stroke, also known as CSVT, is a relatively rare condition with an estimated annual incidence of 7 cases per 1 million neonates [18].

**Fig. 12:**
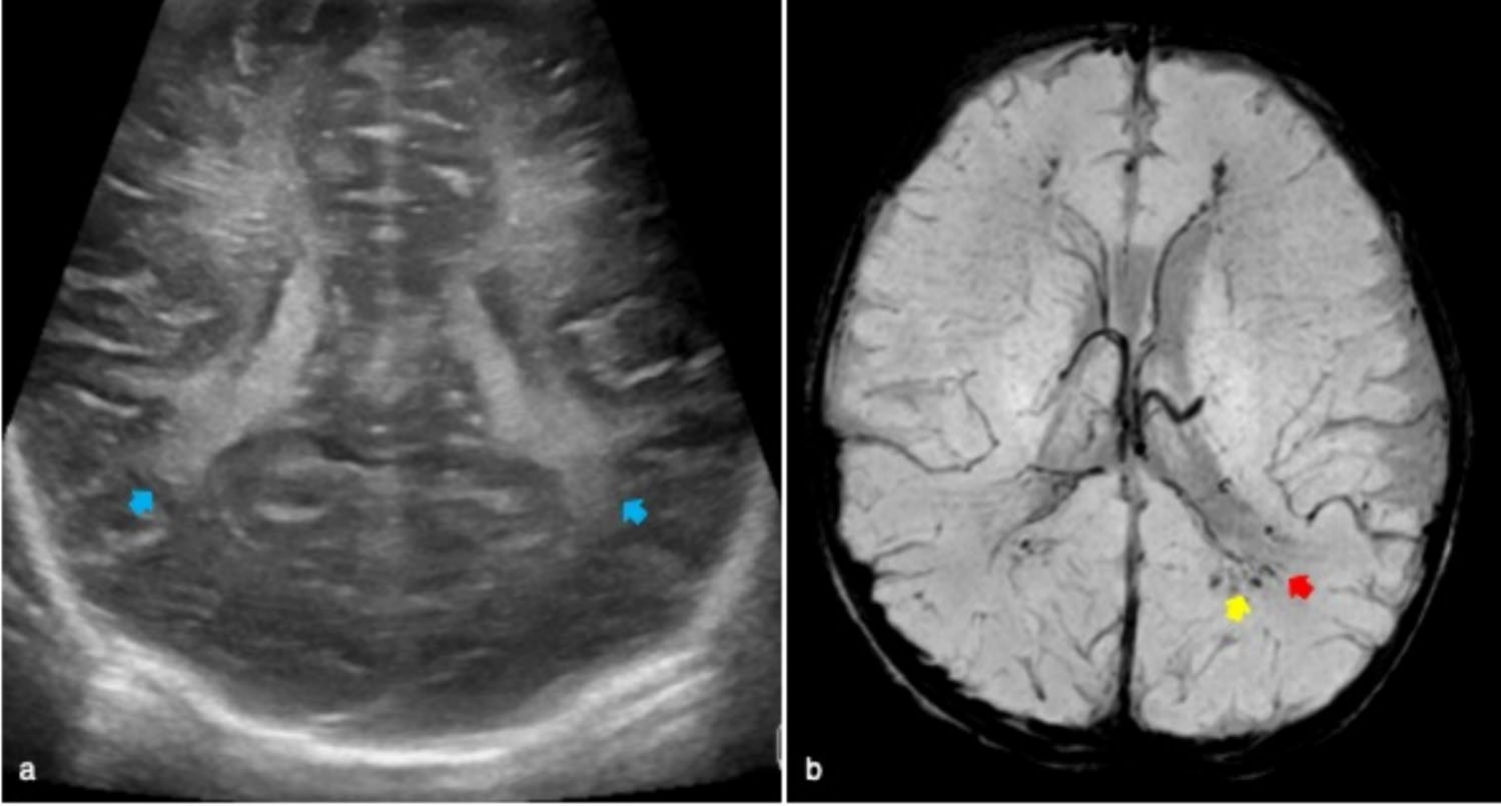
Full term male infant who underwent cerebral ultrasound at 5 days of life for severe perinatal asphyxia requiring neonatal cardio-vascular reanimation. Coronal ultrasound showed fuzzy periventricular hyperechogenic areas (blue arrows in **a**). MR performed to complete brain evaluation well demonstrated periventricular ‘comb’ appearance of the deep medullary veins (red arrow in **b**) and haemorrhagic, punctiform hypointense lesions adjacent to the occipital horns of the lateral ventricles on SWI (yellow arrow in **b**). These findings were suggestive of deep medullary vein thrombosis

**Fig. 13:**
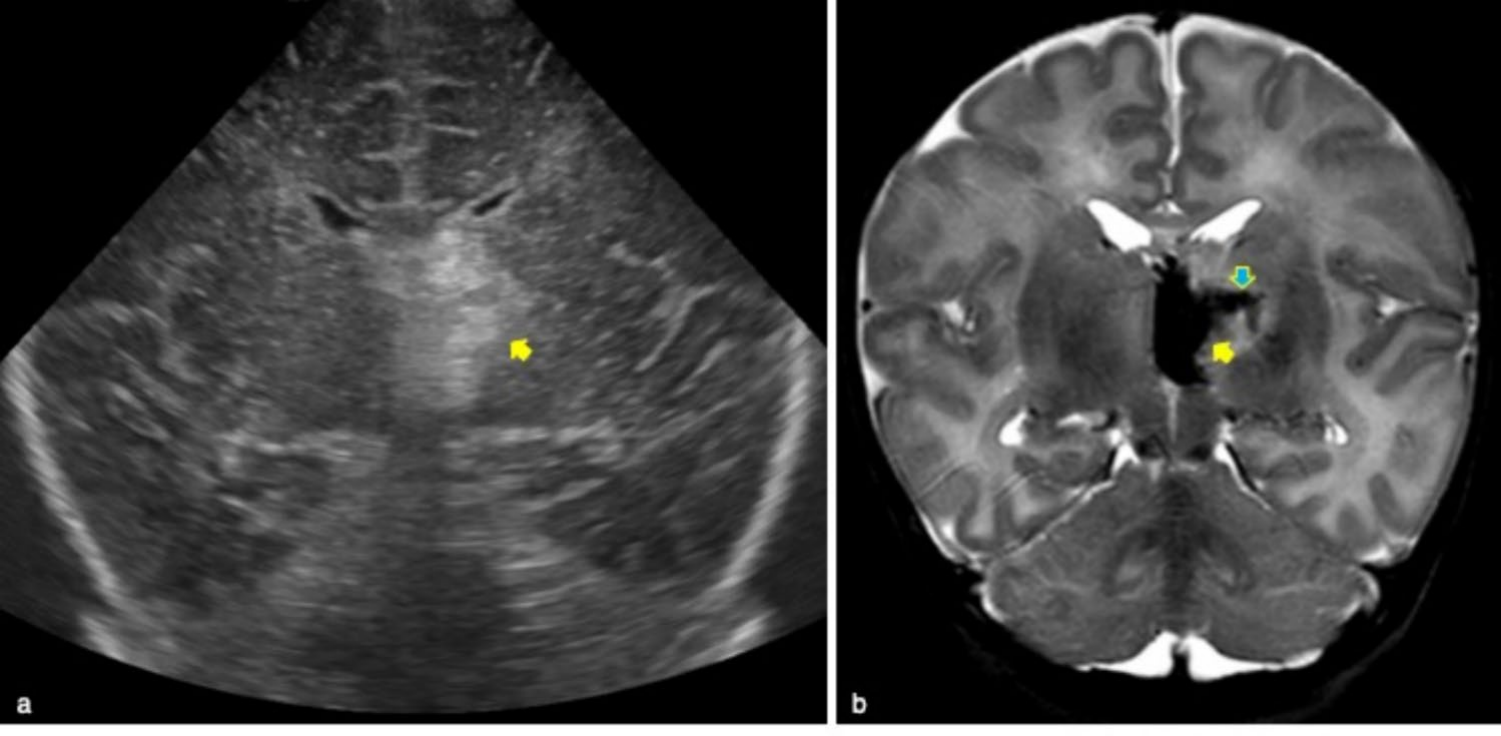
Full term female who underwent radiological examinations at four day of life for hypothermia. Cerebral ultrasound showed left unilateral thalamic echogenic area on coronal plane (yellow arrow in **a**). MR well demonstrated left thalamic hemorrhagic stroke on coronal T2w image (yellow arrow in **b**) associated with linear hypointense area corresponding to thrombosis of thalamic vein (blue arrow in **b**)

It is the result of a combination of risk factors such as infection, dehydration, birth trauma, or genetic disorders. The thrombosis occurs in the cortical/deep cerebral veins rather than in the superficial dural sinuses [19, 20]. If the deep medullary veins are affected an atypical echogenic PVL-like pattern involving the frontal lobes can be seen [19] (Fig. 12). A thalamic hemorrhagic stroke is a further manifestation of a deep cerebral sinus venous thrombosis and it is characterized by an unilateral echogenic lesion in the thalamus and intraventicular hemorrhage often with normal venous flow in the superficial venous system [21] (Fig. 13).

## Older infants

Older infants are included in the range 29 days–2 years.

### Bacterial meningitis

Bacterial meningitis is usually due to the hematogenous or contiguous spread of otitis or sinusitis [1]. In older infants, the most common organism is the Streptococcus Pneumonia. Initially, there is a meningeal infection which involves the choroid plexus and then extends into the cerebrospinal fluid causing a ventriculitis.

**Fig. 14:**
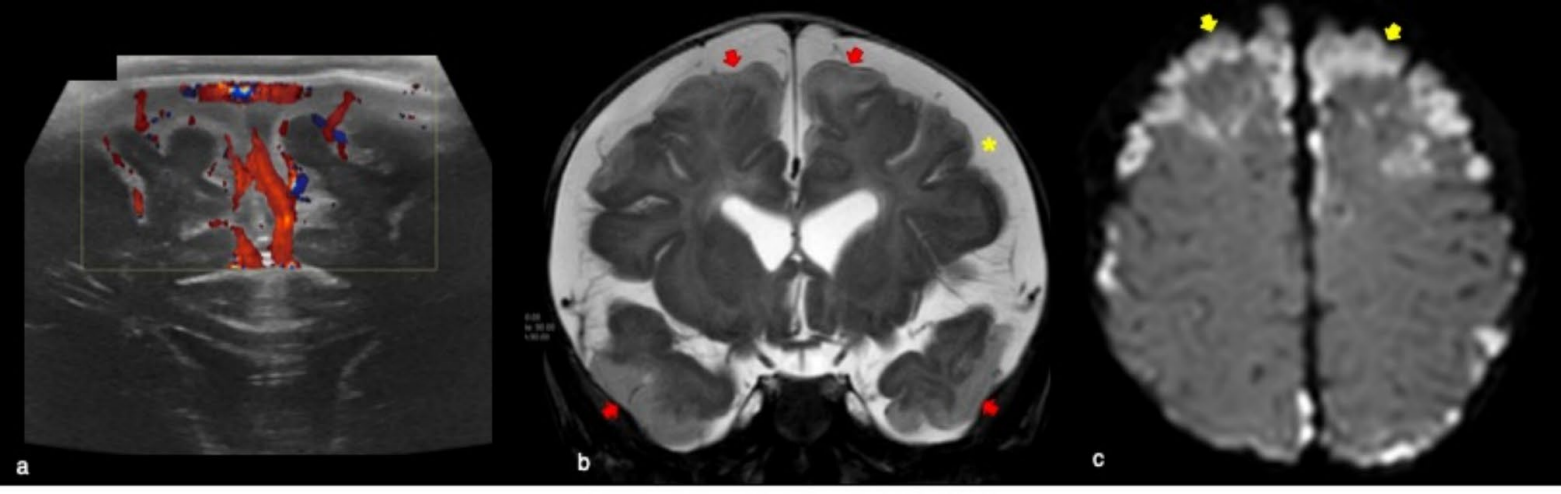
Four-month old male affected by Haemophilus Influenzae meningitis who underwent cerebral ultrasound showing dilatation of extra-axial spaces and increased vascularization on Doppler (**a**). MR of the same patient revealed bilateral subarachnoid empyema in the frontal, temporal and parietal areas on coronal T2 weighted (red arrows in **b**). Bilateral enlargement of supratentorial subarachnoid spaces was also present (yellow asterisk in **b**). DWI showed bilateral subarachnoid empyema and cortico-subcortical areas of ischaemia (yellow arrows in **c**)

The diagnosis of subdural effusion or empyema might not be achieved by cUS but this technique is useful to monitor ventriculitis and ventricular dilatation. The typical features of ventriculitis include intraventricular septa and debris as well as thickening of the ependymal lining (Fig. 14).

### Benign external hydrocephalus (BEH)

**Fig. 15:**
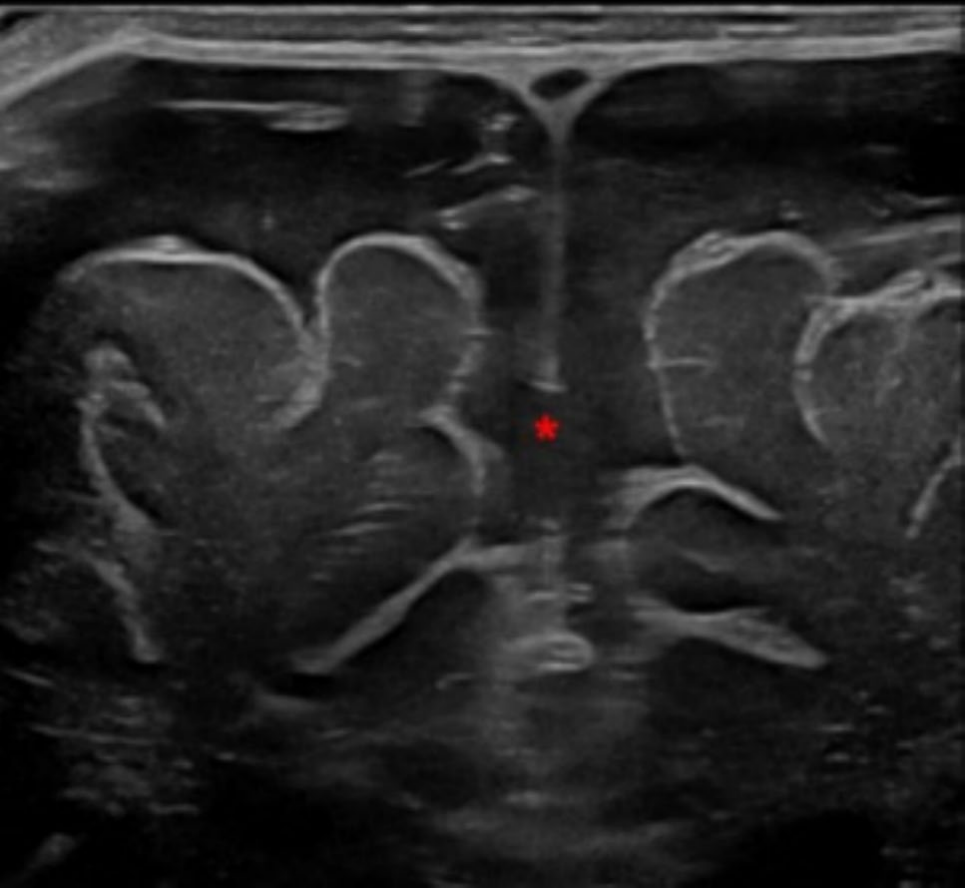
Three month-old male infant who underwent cerebral ultrasound for macrocephaly without other clinical symptoms. Coronal plane showed enlarged extra-axial spaces with increased interhemispheric distance (red asterisk). This finding was suggestive of benign external hydrocephalus

BEH is a common non-progressive condition due to the enlargement of the extra-axial space [22]. Infants show macrocephaly without any other clinical findings. cUS features include bilateral symmetric subarachnoid fluid collections with normal or mildly enlargement of lateral and third ventricles. A consensus about the use of cut-offs for the interhemispheric distance has not been reached yet [23]. By Color Doppler can be seen the veins crossing the subarachnoid space and entering into the superior sagittal sinus [1]. The evaluation of the position of the superior sagittal sinus on the coronal plane allows the distinction between BEH and subdural or epidural collections (Fig. 15).

## Beyond the age-related approach

The age-related classification has some exceptions, in fact several cerebral diseases have no age-group predilection of clinical and/or radiological symptoms occurrence, including for example congenital infection (i.e. TORCH complex), malformations, phacomatoses and tumors. These were not focus of this overview.

## cUS – how to do it and limits

The use of high‑frequency transducer (5–8 MHz) is recommended especially in preterm infants [24]. The anterior fontanelle is the main acoustic window, additional scans through the mastoid fontanelle are also performed to better identify diseases of the posterior cranial fossa. Via the anterior fontanelle six to eight coronal plane images from anterior frontal to the occipital lobes should be obtained. Then five sagittal images, including a midline and two parasagittal views of right and left hemispheres, should be collected [24].

The use of Color-Doppler provides additional information about cerebral perfusion [13]. Despite the advantages carried by cUS, some limitations have to be addressed. For instance, its application in infants older than 3 months is limited due to the closure of the anterior fontanelle. Moreover, MR and CT can provide more accurate anatomical information in specific conditions. For example, CT is beneficial to detect fractures in trauma or extra-axial hemorrhages in suspected physical abuse [25]. MR, on the other hand, is recommended in case perinatal asphyxia, infections (e.g., meningoencephalitis) or tumors.

## Conclusions

In conclusion, cUS is a valuable imaging tool, particularly in neonatal intensive care, enabling a quick and reliable assessment of cerebral variants and diseases. A deep knowledge of the typical cUS findings, in association with the age of the infant, can provide an essential support to pediatricians and radiologists in achieving the correct diagnosis.

## Data Availability

Not applicable.
